# Postnatal Ablation of Foxm1 from Cardiomyocytes Causes Late Onset Cardiac Hypertrophy and Fibrosis without Exacerbating Pressure Overload-Induced Cardiac Remodeling

**DOI:** 10.1371/journal.pone.0048713

**Published:** 2012-11-08

**Authors:** Craig Bolte, Yufang Zhang, Allen York, Tanya V. Kalin, Jo El J. Schultz, Jeffery D. Molkentin, Vladimir V. Kalinichenko

**Affiliations:** 1 Division of Pulmonary Biology, Cincinnati Children's Hospital Research Foundation, Cincinnati, Ohio, United States of America; 2 Molecular Cardiovascular Biology, Cincinnati Children's Hospital Research Foundation, Cincinnati, Ohio, United States of America; 3 Department of Pharmacology and Cell Biophysics, University of Cincinnati College of Medicine, Cincinnati, Ohio, United States of America; Northwestern University, United States of America

## Abstract

Heart disease remains a leading cause of morbidity and mortality in the industrialized world. Hypertrophic cardiomyopathy is the most common genetic cardiovascular disorder and the most common cause of sudden cardiac death. Foxm1 transcription factor (also known as HFH-11B, Trident, Win or MPP2) plays an important role in the pathogenesis of various cancers and is a critical mediator of post-injury repair in multiple organs. Foxm1 has been previously shown to be essential for heart development and proliferation of embryonic cardiomyocytes. However, the role of Foxm1 in postnatal heart development and in cardiac injury has not been evaluated. To delete *Foxm1* in postnatal cardiomyocytes, *αMHC-Cre/Foxm1^fl/fl^* mice were generated. Surprisingly, *αMHC-Cre/Foxm1^fl/fl^* mice exhibited normal cardiomyocyte proliferation at postnatal day seven and had no defects in cardiac structure or function but developed cardiac hypertrophy and fibrosis late in life. The development of cardiomyocyte hypertrophy and cardiac fibrosis in aged Foxm1-deficient mice was associated with reduced expression of Hey2, an important regulator of cardiac homeostasis, and increased expression of genes critical for cardiac remodeling, including MMP9, αSMA, fibronectin and vimentin. We also found that following aortic constriction Foxm1 mRNA and protein were induced in cardiomyocytes. However, *Foxm1* deletion did not exacerbate cardiac hypertrophy or fibrosis following chronic pressure overload. Our results demonstrate that Foxm1 regulates genes critical for age-induced cardiomyocyte hypertrophy and cardiac fibrosis.

## Introduction

Cardiac hypertrophy can result from increased hemodynamic load, myocardial infarction, extreme athletic training, aging, chronic respiratory diseases or can be seemingly idiopathic. Hypertrophy following an increased hemodynamic load is a compensatory mechanism to maintain perfusion of the peripheral tissues and prevent cardiac insufficiency. However, cardiac remodeling is only compensatory in the short term and leads to heart failure when either the chamber walls become too thin and weak to effectively expel blood (systolic dysfunction) or when thickening of the myocardium decreases the lumen of the chamber to the point the heart can no longer fill properly (diastolic dysfunction). Both outcomes are irreversible and require surgical intervention. Idiopathic cardiac hypertrophy typically manifests from a genetic predisposition and is known as hypertrophic cardiomyopathy. Hypertrophic cardiomyopathy (HCM) is defined as hypertrophy of the myocardium in the absence of sufficient external force [1], [2]. Hundreds of mutations in at least 13 known genes, mostly sarcomeric proteins, have been linked to HCM [2]; however, mutations in genes encoding proteins of the extracellular matrix can produce a similar phenotype [3]–[5]. With a frequency of 1 in 500 in the general population, HCM is the most common genetic cardiovascular disorder [2] and also the most common cause of sudden cardiac death in individuals under 35 years of age [2]. In the absence of family history HCM often goes undiagnosed until cardiac arrhythmias occur, potentially culminating in sudden cardiac death. In many cases HCM is discovered by electrocardiography for another indication, with echocardiography remaining the best means to evaluate the extent and track the progression of HCM [2].

**Table 1:** TaqMan gene expression assays (Applied Biosystems) used for qRT-PCR analysis.

Mouse α-SMA	Mm00702100_s1
Mouse β-actin	Mm00607939_s1
Mouse BMP4	Mm01321704_m1
Mouse CaMKIIδ	Mm00499266_m1
Mouse CDC25B	Mm00499136_m1
Mouse collagen 1α1	Mm00801666_g1
Mouse collagen 3α1	Mm00802331_m1
Mouse CXCR4	Mm01292123_m1
Mouse Cyclin B_1_	Mm00838401_g1
Mouse Fibronectin	Mm01256744_m1
Mouse Foxm1	Mm00514924_m1
Mouse Fsp-1	Mm00724330_m1
Mouse Hey2	Mm00469280_m1
Mouse IL-1β	Mm01336189_m1
Mouse IL-1R	Mm01337566_m1
Mouse IκBKB	Mm01222247_m1
Mouse MMP2	Mm00439498_m1
Mouse MMP9	Mm00442991_m1
Mouse Myocardin	Mm00455051_m1
Mouse NFATc3	Mm01249200_m1
Mouse NFκB	Mm00479807_m1
Mouse nMyc	Mm00476449_m1
Mouse p21^cip1^	Mm01303209_m1
Mouse Plk-1	Mm00440924_g1
Mouse TGF-β1	Mm01178820_m1
Mouse TIMP1	Mm00441818_m1
Mouse TNFα	Mm00443258_m1
Mouse Vimentin	Mm00449201_m1

Foxm1 (formerly known as HFH-11B, Trident, Win or MPP2) is a member of the Forkhead Box (Fox) family of transcription factors that share homology in the winged helix/forkhead DNA binding domain. Foxm1 is highly expressed in proliferating cells, but expression wanes postnatally and Foxm1 protein can only be detected in intestinal crypts, testes and thymus in the adult [6], [7]. Foxm1 expression in human fibroblasts has been previously shown to decrease with advancing age and to be decreased in Progeria syndrome, which is characterized by premature aging [8]. Overexpression of Foxm1 in transgenic mice prevented age-related defects during liver repair, implicating Foxm1 in regulation of the aging process [9]. We have previously shown that Foxm1 plays an essential role in embryonic cardiac development [10], [11]. Ablation of *Foxm1* from all cell types (*Foxm1^−/−^*) [11], [12] or cardiomyocyte-specific deletion of Foxm1 (*Nkx2.5-Cre/Foxm1^fl/fl^*) [10] is embryonic lethal due to defects in cardiomyocyte proliferation. Cardiomyocytes from *Foxm1^−/−^* and *Nkx2.5-Cre/Foxm1^fl/fl^* mice were enlarged and the percentage of proliferating cardiomyocytes was decreased. Deletion of *Foxm1* in embryonic cardiomyocytes caused early withdrawal from the cell cycle, resulting in premature entrance into the hypertrophic phase of cardiac growth. Due to embryonic lethality in *Foxm1^−/−^* and *Nkx2.5-Cre/Foxm1^fl/fl^* mice, the role of Foxm1 in postnatal cardiac development and cardiac injury remains unknown.

**Figure 1:**
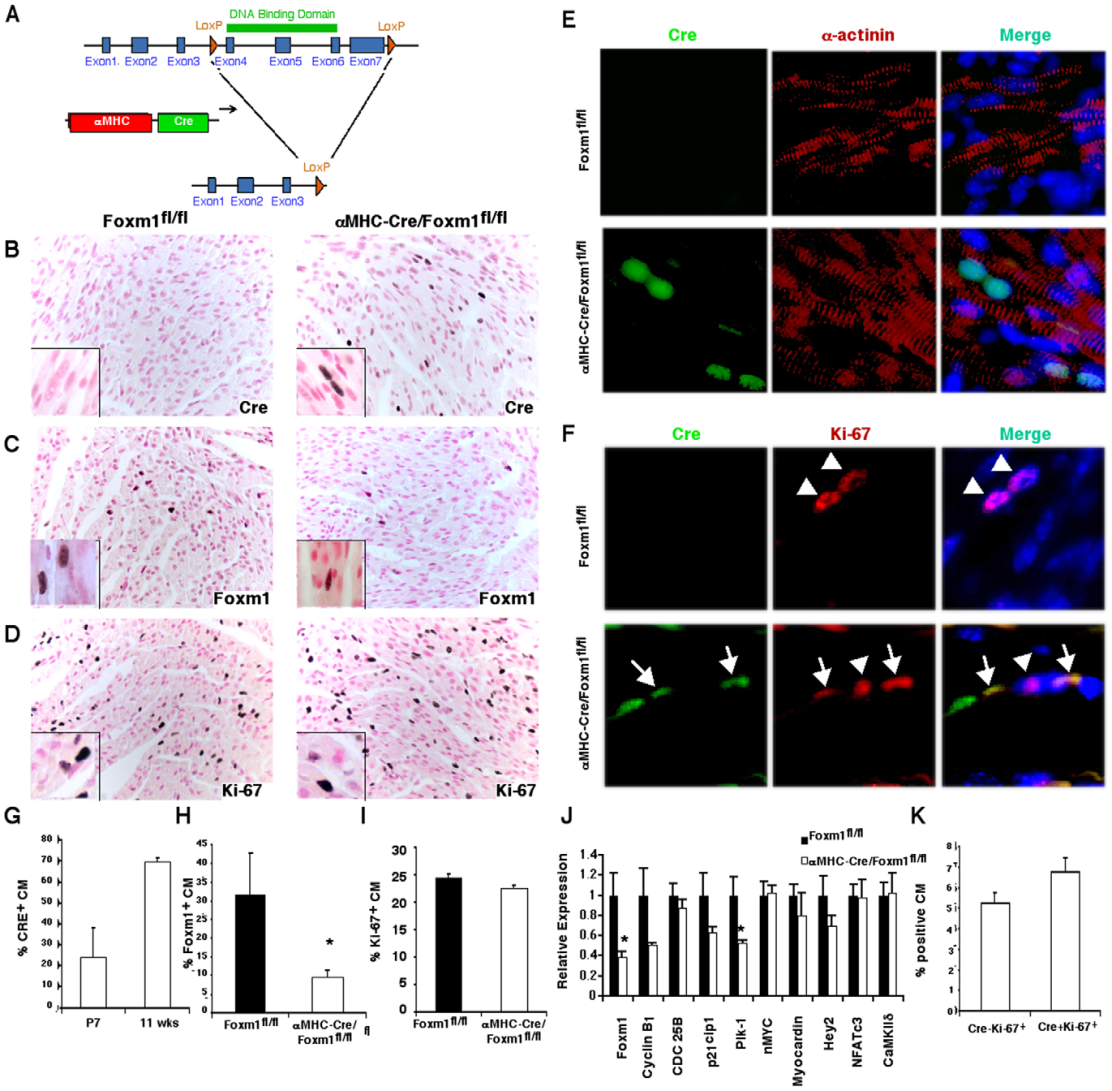
Cardiomyocyte proliferation unaltered at P7 in α*MHC-Cre/Foxm1^fl/fl^* mice. The Cre-LoxP system was utilized to conditionally delete *Foxm1* from cardiomyocytes (CM). Mice homozygous for the *Foxm1^fl/fl^* allele (in which LoxP sites flank exons 4 through 7 of the *Foxm1* gene) were mated to mice expressing *αMHC-Cre* to generate mice with a conditional deletion of *Foxm1* from cardiomyocytes (*αMHC-Cre/Foxm1^fl/fl^*) (A). Cre expression was observed in approximately 25% of cardiomyocytes at postnatal day 7 (P7) in *αMHC-Cre/Foxm1^fl/fl^* mice (G). Cre was not detected in control hearts (B, E). The number of FOXM1-positive cardiomyocytes was higher in *Foxm1^fl/fl^* than in *αMHC-Cre/Foxm1^fl/fl^* mice at P7 (C, H). Ki-67 staining was similar in *αMHC-Cre/Foxm1^fl/fl^* and control hearts (D, I). There was no difference in the percentage of *αMHC-Cre/Foxm1^fl/fl^* cardiomyocytes positive for Ki-67 (Cre^−^Ki-67^+^) (arrow heads) and those double positive for Ki-67 and Cre (Cre^+^Ki-67^+^) (arrows) (F, K). Foxm1 mRNA was decreased in *αMHC-Cre/Foxm1^fl/fl^* hearts as was Plk-1. Cyclin B_1_, CDC 25B, p21^cip1^, nMYC, myocardin, Hey2, NFATc3 and CaMKIIδ mRNAs were unaltered (J). Significant differences (p<0.05) were indicated by asterisk. “N” was 4 for all groups. Magnifications were 40x (B–D), 400x (insets B–D) and 200x (E–F).

In this study, we generated a new mouse line with cardiomyocyte-specific Foxm1 deletion after birth (*αMHC-Cre/Foxm1^fl/fl^*). Surprisingly, hearts from these mice were structurally and functionally normal, indicating that Foxm1 is dispensable for postnatal heart development. *αMHC-Cre/Foxm1^fl/fl^* mice developed cardiac hypertrophy and fibrosis after aging. The development of cardiac fibrosis and cardiomyocyte hypertrophy in aged *αMHC-Cre/Foxm1^fl/fl^* mice was associated with reduced expression of Hey2, a critical regulator of cardiac homeostasis [13]. Given the vital role Foxm1 plays during cardiac embryogenesis as well as the predisposition for end of life cardiac hypertrophy in *αMHC-Cre/Foxm1^fl/fl^* mice, we also investigated the importance of Foxm1 in cardiac pathology using a mouse model of cardiac hypertrophy induced by aortic banding. Although Foxm1 protein and message were increased following transaortic constriction, deletion of Foxm1 from cardiomyocytes did not affect survival, the development of cardiac hypertrophy or the degree of cardiac remodeling. Thus, Foxm1 expression in cardiomyocytes is critical for age-related cardiac hypertrophy but dispensable for pressure overload-induced cardiomyocyte hypertrophy.

**Figure 2:**
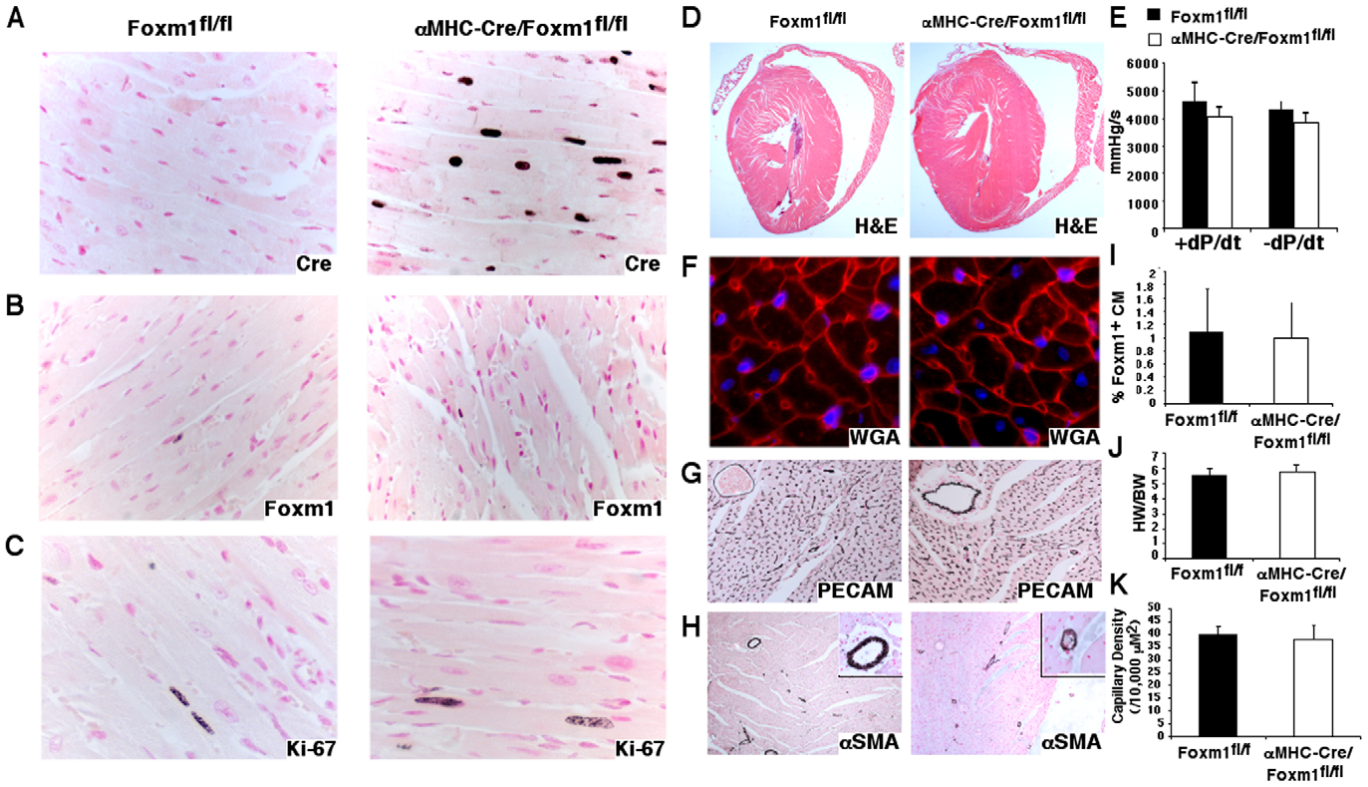
Postnatal deletion of Foxm1 does not alter cardiac morphology or function. Immunohistochemistry showed Cre protein in adult *αMHC-Cre/Foxm1^fl/fl^* cardiomyocytes (A). FOXM1-positive cardiomyocytes were rare in adult *αMHC-Cre/Foxm1^fl/fl^* and control mice (B, I), as were Ki-67-positive cells (C). Hearts from adult *αMHC-Cre/Foxm1^fl/fl^* mice were similar in size to *Foxm1^fl/fl^* control mice (D, J) and had similar cardiac inotropy (+dP/dt) and lusitropy (−dP/dt) (E). There was no change in cardiomyocyte size as indicated by WGA staining (F), capillary density as shown by PECAM-1 staining (G, K) or coronary artery formation as visualized by αSMA staining (H). “N” was 5 control and 6 *αMHC-Cre/Foxm1^fl/fl^* mice. Magnifications were (A–C, F, inset H) 40x, (G & H) 20x and (D) 5x.

## Materials and Methods

### Mice

We have previously described the generation of *Foxm1* LoxP/LoxP (*Foxm1^fl/fl^*) mice, in which LoxP sequences flank exons 4 through 7 of the *Foxm1* gene that encode the DNA binding and transcriptional activation domains of the FOXM1 protein [14]. *Foxm1^fl/fl^* mice were bred with *αMHC-Cre* mice [15] to generate *αMHC-Cre/Foxm1^fl/fl^* mice, resulting in postnatal cardiomyocyte-specific deletion of *Foxm1*. *αMHC-Cre/Foxm1^fl/fl^* mice were bred with *Foxm1^fl/fl^* mice to generate litters with an expected 1∶1 ratio of *αMHC-Cre/Foxm1^fl/fl^* mice to *Foxm1^fl/fl^* control mice.

**Figure 3:**
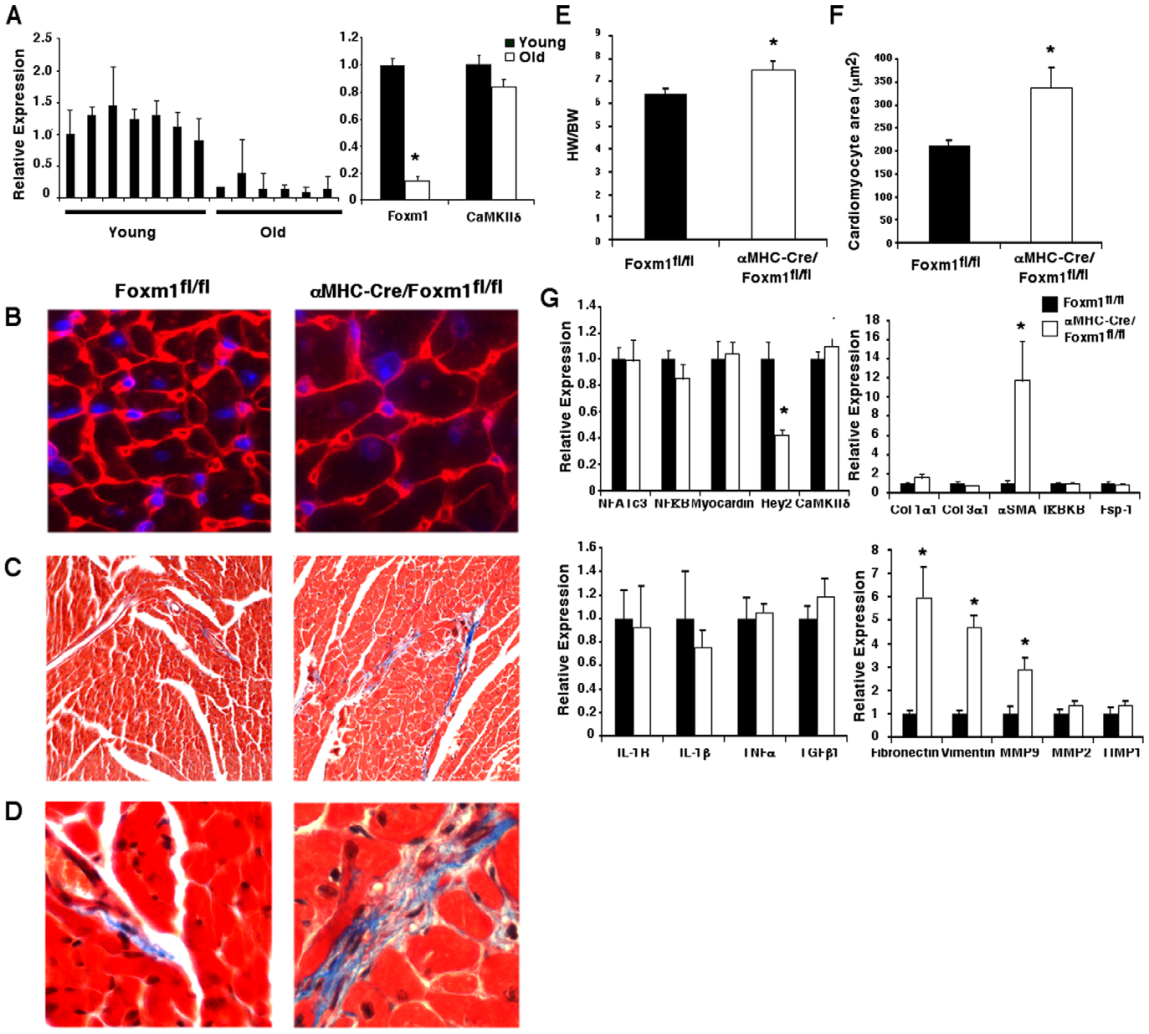
Cardiac hypertrophy and fibrosis in aged α*MHC-Cre/Foxm1^fl/fl^* mice. Foxm1 mRNA was significantly decreased, while CaMKIIδ mRNA was unaltered, in hearts of old WT mice compared to young mice as shown by qRT-PCR (A). Old *αMHC-Cre/Foxm1^fl/fl^* mice had significant cardiac hypertrophy as indicated by increased heart weight-to-body weight ratios (E) and increased cardiomyocyte area (F). Cardiomyocyte area was visualized and measured in WGA stained sections using 50 cardiomyocytes from random fields (B). Cardiac fibrosis was increased in old *αMHC-Cre/Foxm1^fl/fl^* mice as shown by Masson's Trichrome staining (C, D). qRT-PCR analysis showed increased αSMA, fibronectin, vimentin and MMP9 and decreased Hey2 mRNA levels in hearts of old *αMHC-Cre/Foxm1^fl/fl^* mice compared to age-matched control mice (G). Significant differences (p<0.05) were indicated by asterisk. “N” was 7 young and 6 old wildtype mice (A) and 6 control and 4 *αMHC-Cre/Foxm1^fl/fl^* mice (E–G). Magnifications were 40x (B, D) and 10x (C).

### Ethics statement

Animal studies were reviewed and approved by the Animal Care and Use Committee of Cincinnati Children's Hospital Research Foundation (protocol # 0D08057).

**Figure 4:**
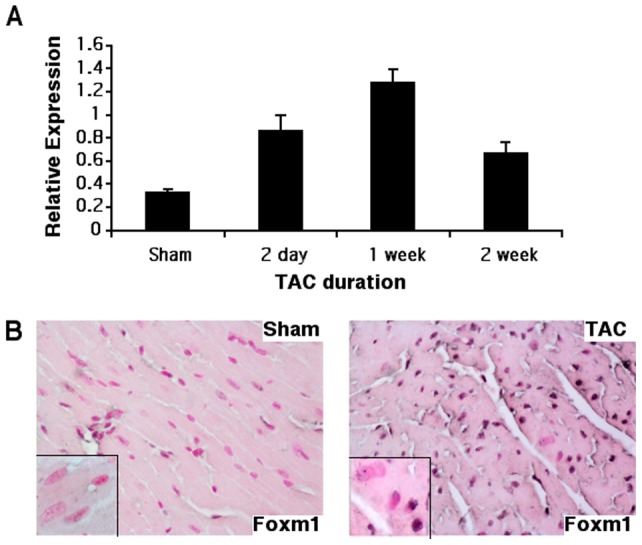
Foxm1 mRNA and protein increased following aortic banding. qRT-PCR showed increased whole-heart Foxm1 mRNA expression as early as 2 days after TAC surgery with levels peaking around a week post-surgery and remaining elevated for at least 2 weeks (A). The incidence of FOXM1-positive cardiomyocytes was increased 2 weeks after aortic banding as shown by immunostaining with FOXM1-specific antibodies (B). Magnifications were 40x (B) and 400x (B insets).

### Evaluation of cardiac function

*In vivo* cardiac function was determined in *αMHC-Cre/Foxm1^fl/fl^* and control mice not undergoing surgery by Millar catheterization as described [16]. Briefly, following anesthesia a 2F Millar Catheter was inserted into the lumen of the carotid artery and advanced until it reached the left ventricle of the heart. Once inside the left ventricle, the catheter was held in place with suture. Interventricular pressure was recorded with a DigiMed system recorder. A 3-point EKG was attached to the mouse and sinus rhythm recorded with DigiMed software.

**Figure 5:**
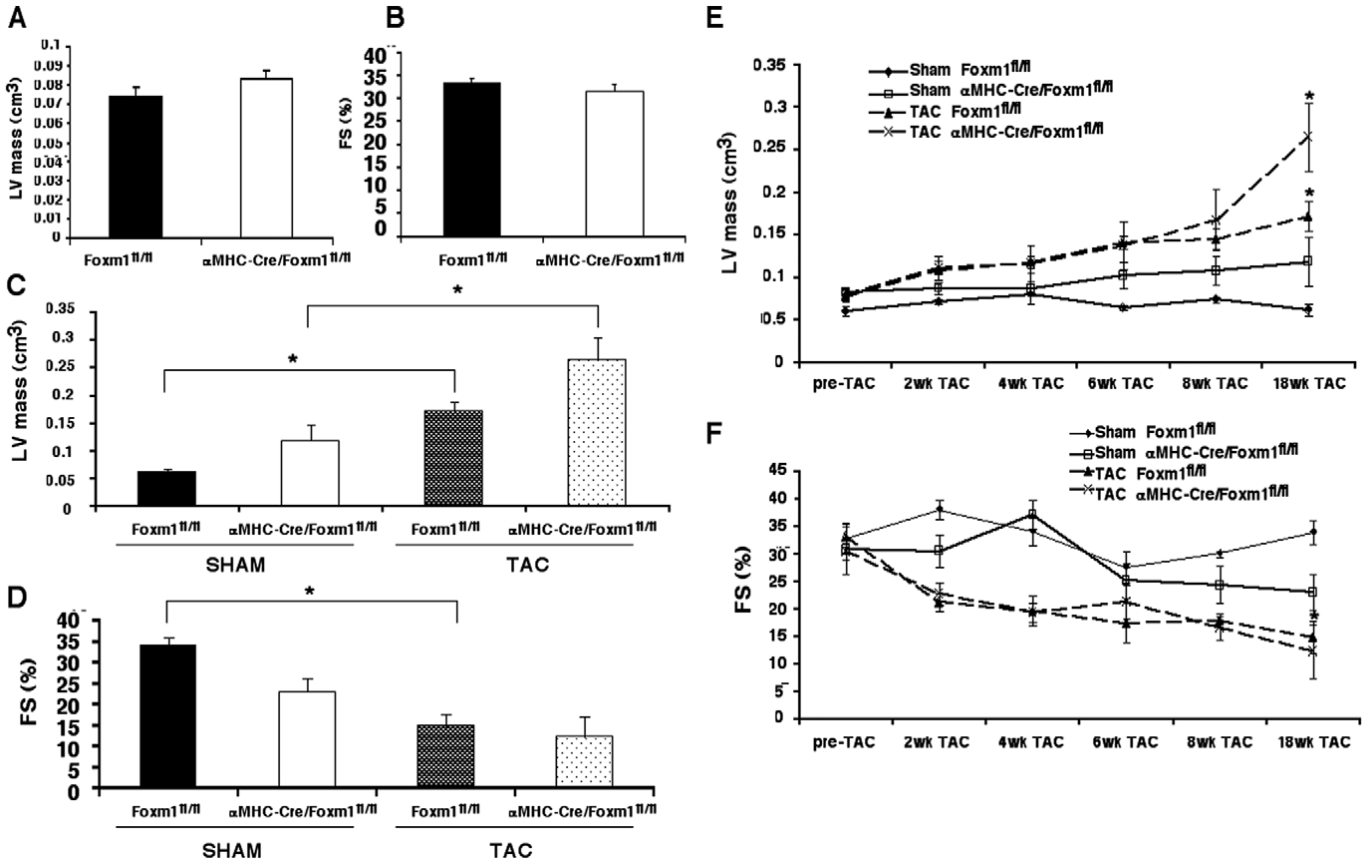
Foxm1 deficiency does not affect TAC-induced cardiac hypertrophy. *Foxm1^fl/fl^* and *αMHC-Cre/Foxm1^fl/fl^* mice were evaluated by echocardiography prior to aortic banding and serially throughout the development of cardiac hypertrophy. There was no difference between *Foxm1^fl/fl^* and *αMHC-Cre/Foxm1^fl/fl^* mice in LV mass (A) or fractional shortening (FS) prior to aortic banding (B). Aortic banding caused a significant increase in LV mass in mice from both lines without a difference between *Foxm1^fl/fl^* and *αMHC-Cre/Foxm1^fl/fl^* mice (C, E). Fractional shortening was decreased following aortic banding with no difference between lines (D, F). Sham surgery mice were used as control. Significant differences between TAC and Sham treated mice (p<0.05) were indicated by asterisk. “N” was 3 for all groups.

**Figure 6:**
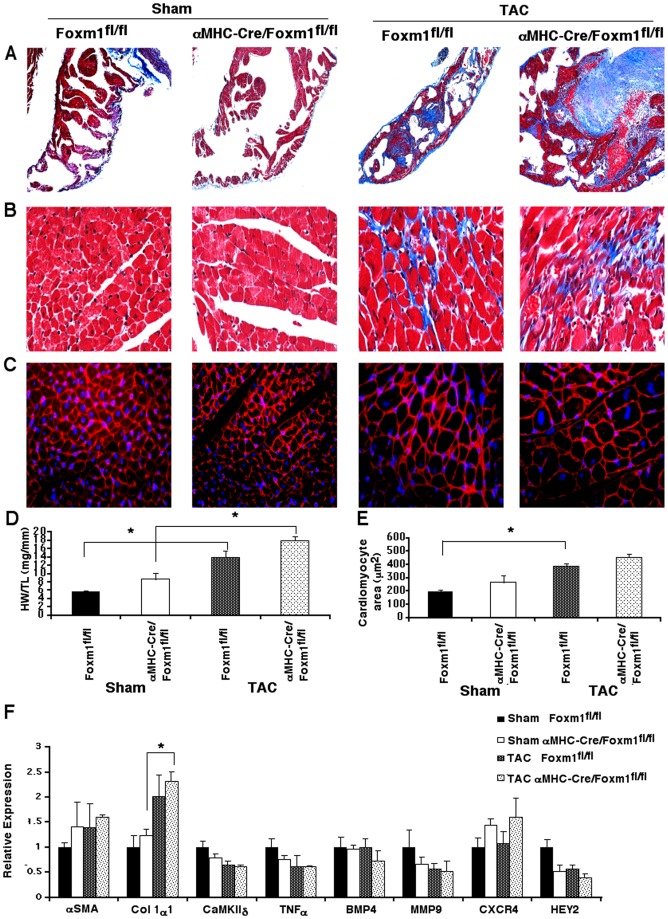
Cardiac remodeling is unaltered following TAC surgery in α*MHC-Cre/Foxm1^fl/fl^* mice. Masson's Trichrome staining of mice 18 weeks after aortic banding indicated fibrotic deposition in the atria (A) and ventricles (B) was similar between *αMHC-Cre/Foxm1^fl/fl^* and control mice. WGA staining showed cardiomyocyte size significantly increased following aortic banding with no difference between mouse lines (C, E). Heart weight-to-tibia length ratios were unaltered between *αMHC-Cre/Foxm1^fl/fl^* and control mice following TAC surgery (D). qRT-PCR showed an increase in collagen 1α1 mRNA following TAC (F). mRNA levels of αSMA, CaMKIIδ, TNFα, BMP4, MMP9, CXCR4 and Hey2 were unaltered. Significant differences (p<0.05) were indicated by asterisk. “N” was 3 for all groups. Magnifications were (A) 5x and (B, C) 20x.

### Aortic banding

Cardiac hypertrophy was induced in *αMHC-Cre/Foxm1^fl/fl^* and control *Foxm1^fl/fl^* mice by aortic banding as described [17]. Banding was performed on mice of both sexes at 8 to 10 weeks of age. No differences were observed between males and females. Mice were sedated with 3% isoflurane and 600 mL/min O_2_. Subsequently, mice were intubated to maintain pulmonary pressure, the sternum split, ribs retracted and the left portion of the thymus removed to allow aortic visualization. To ensure a reproducible degree of aortic constriction, suture was secured around a bent 27G needle between the 1^st^ and 2^nd^ branches of the aortic arch. Once the suture was in place, the needle was removed, the thoracic cavity was closed and the chest evacuated to prevent a pneumothorax.

### Echocardiography

Progression of cardiac hypertrophy was monitored by echocardiography as described [17]. Mice from all genotypes and treatment groups were anesthetized with isoflurane and echocardiography was performed using a SONOS 5500 instrument (Hewlett Packard) with a 15-MHz microprobe. Echocardiographic measurements were taken on M-mode in triplicate for each mouse at the level of the papillary muscle. Pressure gradients across the constriction were measured by Doppler echocardiography. Fractional shortening was calculated as ([LVEDdLVEDs]/LVEDd)×100, (%) [18].

### Immunohistochemical staining

Hearts from *αMHC-Cre/Foxm1^fl/fl^* and control *Foxm1^fl/fl^* mice were collected at postnatal day 7 (P7), 11 weeks of age and 16 months of age. Aortic constriction was performed on 8–10 week old mice and mice were harvested 18 weeks after aortic banding. Hearts were fixed in 4% paraformaldehyde overnight and embedded into paraffin blocks. Paraffin sections of 5 µm were immunostained for FOXM1 (1∶500; k-19; Santa Cruz), Cre (1∶5000; 7 Hills Biotech), PECAM-1 (1∶10,000; Pharminogen), α-smooth muscle actin (αSMA; 1∶10,000; Sigma-Aldrich) or Ki-67 (1∶10,000; Dako). Antibody-antigen complexes were detected using biotinylated secondary antibody followed by avidin-HRP complex and DAB substrate (Vector Labs, Burlingame, CA), as previously described [11], [19]–[21]. Sections were counterstained with nuclear fast red. Co-localization studies were performed using primary antibodies against α-actinin (1∶100; Sigma-Aldrich), Ki-67 (1∶400), FOXM1 (1∶50) and Cre (1∶500) followed by secondary antibodies labeled with fluorescein isothiocynate (FITC) or Texas Red. Slides were counterstained with DAPI to detect cell nuclei (Vector Labs). Heart sections were also stained with hematoxylin and eosin (H&E) to evaluate cardiac morphology, wheat-germ agglutinin to measure cardiomyocyte size or Masson's Trichrome to detect cardiac fibrosis. Slides were photographed using a Zeiss Axioplan2 microscope and Axiovision Rel 4.8 software.

### Quantitative real-time RT-PCR (qRT-PCR)

Total cardiac RNA was prepared from individual *αMHC-Cre/Foxm1^fl/fl^* and control *Foxm1^fl/fl^* hearts using RNA-STAT-60 (Tel-Test “B” Inc. Friendswood, TX). cDNA was generated using the Applied Biosystems High Capacity cDNA Reverse Transcription kit (Applied Biosystems, Foster City, CA). Evaluation of expression levels of specific genes was performed by qRT-PCR using Taqman probes (Table 1) and the StepOnePlus Real-Time PCR system (Applied Biosystems, Foster City, CA), as previously described [19], [22], [23].

### Statistical analysis

Student's T-test was used to determine statistical significance. P values <0.05 were considered significant. Values for all measurements were expressed as mean ± standard error of mean (SEM).

## Results

### Cardiomyocyte proliferation unaltered at P7 in *αMHC-Cre/Foxm1^fl/fl^* mice

*αMHC-Cre* transgenic mice [15] were bred with *Foxm1^fl/fl^* mice [14] to produce *αMHC-Cre/Foxm1^fl/fl^* mice. In these mice, exons 4–7 encoding the DNA binding and transcriptional activation domains of the FOXM1 protein were deleted specifically from postnatal cardiomyocytes (Figure 1A). At P7, approximately 25% of cardiomyocytes in *αMHC-Cre/Foxm1^fl/fl^* mice were positive for Cre protein (Figure 1B & G). Cre-expressing cardiomyocytes maintained expression of α-actinin, a sarcomeric marker (Figure 1E). Decreased expression of Foxm1 mRNA (Figure 1J) as well as less FOXM1-positive cardiomyocytes was observed in *αMHC-Cre/Foxm1^fl/fl^* hearts at P7 when compared to age-matched controls (Figure 1C & H). Despite decreased FOXM1 protein and mRNA in juvenile cardiomyocytes, there was no difference in the percentage of Ki-67-positive cells at P7 (Figure 1D & I). Co-localization experiments showed that cardiomyocytes positive for Cre expressed the proliferation-specific protein Ki-67 (Figure 1F), indicating they undergo the cell cycle. The percentage of proliferating cells was similar in Cre-positive and Cre-negative subsets of *αMHC-Cre/Foxm1^fl/fl^* cardiomyocytes (Figure 1F & K). Deletion of *Foxm1* in postnatal cardiomyocytes reduced mRNA of Plk-1, a known Foxm1 target gene [10], [24], but did not significantly influence mRNA levels of cyclin B_1_, CDC 25B, p21^cip1^, nMYC, myocardin, Hey2, NFATc3 or CaMKIIδ (Figure 1J). Thus *Foxm1* deletion in *αMHC-Cre/Foxm1^fl/fl^* mice does not influence proliferation of cardiomyocytes in P7 hearts.

### Normal cardiac morphology and function in adult *αMHC-Cre/Foxm1^fl/fl^* mice

Cre expression was considerably higher in cardiomyocytes from adult (10–13 weeks) *αMHC-Cre/Foxm1^fl/fl^* mice compared to P7 mice (Figures 1B & G & 2A). Few cardiomyocytes stained positively for FOXM1 in either line at this time point (Figure 2B & I), and there was no change in cardiomyocyte proliferation as demonstrated by Ki-67 staining (Figure 2C). Histological evaluation of *αMHC-Cre/Foxm1^fl/fl^* mice showed no gross morphological alterations in right or left ventricular anatomy compared to control *Foxm1^fl/fl^* littermates (Figure 2D) and heart weight-to-body weight ratios were similar (Figure 2J). Cardiomyocyte size (Figure 2F), capillary density (Figure 2G & K) and coronary vessel formation (Figure 2H) were also normal in *αMHC-Cre/Foxm1^fl/fl^* mice. Furthermore, *in vivo* parameters of cardiac inotropy (+dP/dt) and lusitropy (−dP/dt) were indistinguishable between adult *αMHC-Cre/Foxm1^fl/fl^* and control mice (Figure 2E). Nor was there a difference in sinus rhythm or greater occurrence of arrhythmia in *αMHC-Cre/Foxm1^fl/fl^* compared to control mice (data not shown). Thus, postnatal deletion of *Foxm1* does not influence heart structure or function in adult mice.

### Late onset cardiac hypertrophy in old *αMHC-Cre/Foxm1^fl/fl^* mice

Foxm1 mRNA levels decreased in hearts of old (16 month) WT mice compared to young adult mice, while CaMKIIδ remained constant (Figure 3A). Cardiac structure was altered in old *αMHC-Cre/Foxm1^fl/fl^* mice. Consistent with late onset cardiac hypertrophy, old *αMHC-Cre/Foxm1^fl/fl^* mice had a significant increase in heart weight-to-body weight ratio compared to control mice (Figure 3E). Cardiomyocyte size in both ventricles in *Foxm1*-deficient hearts was increased by 60% as demonstrated by staining of heart sections with wheat-germ agglutinin (Figure 3B & F). In addition, increased myocardial fibrosis was observed by Masson's Trichrome staining in old *αMHC-Cre/Foxm1^fl/fl^* hearts compared to age-matched controls (Figure 3C & D). Real-time RT-PCR analysis showed a decrease in Hey2 mRNA (Figure 3G), a transcription factor implicated in the development of cardiomyocyte hypertrophy [25], [26]. Expression of genes critical for cardiac fibrosis, such as αSMA, fibronectin, vimentin and MMP9 was significantly increased in old *αMHC-Cre/Foxm1^fl/fl^* hearts compared to age-matched controls. mRNAs of NFκB, NFATc3, myocardin, IL-1β, IL-1Rα, TNFα, TGFβ, MMP2, TIMP1, Fsp-1, IκBKB and CaMKIIδ were unaltered (Figure 3G). Thus Foxm1 is critical for proper maintenance of myocardial structure during aging.

### Foxm1 expression in cardiomyocytes is not critical for TAC-induced cardiac hypertrophy

Experiments were performed to examine Foxm1 expression during pathological cardiac hypertrophy. Following aortic constriction in young WT mice, increased Foxm1 mRNA was observed as soon as 2 days and elevated levels were maintained for 14 days after injury (Figure 4A). FOXM1 protein was increased in several cell types including cardiomyocytes, endothelial and inflammatory cells 2 weeks after banding (Figure 4B and data not shown).

We next determined the specific role of Foxm1 in cardiomyocytes during TAC-mediated hypertrophy. *αMHC-Cre/Foxm1^fl/fl^* and control mice at 8–10 weeks old were subjected to echocardiography prior to aortic banding as well as serially throughout the subsequent progression of cardiac hypertrophy. Based on echocardiography, there were no differences in left ventricular (LV) mass or fractional shortening between *αMHC-Cre/Foxm1^fl/fl^* and control mice prior to aortic banding (Figure 5A & B). Aortic banding caused a significant increase in minimum and maximum aortic pressure in all mice, with no difference between control and *αMHC-Cre/Foxm1^fl/fl^* mice (58.6±6.2 mmHg vs. 48.4±4.9 mmHg pressure gradient across constriction, respectively). Increased aortic pressure equally induced cardiac hypertrophy in both groups of mice, as evidenced by increased LV mass (Figure 5C & E), and decreased cardiac function, indicated by fractional shortening (Figure 5D & F). Heart weight-to-tibia length ratios (Figure 6D) and cardiomyocyte area (Figure 6C & E) were similar in *αMHC-Cre/Foxm1^fl/fl^* and control mice. Cardiac fibrosis, as shown by Masson's Trichrome staining, was also similar between *Foxm1*-deficient and control mice (Figure 6A and B). *Foxm1* deletion did not alter mRNA expression of genes critical for cardiac hypertrophy and fibrosis such as α-SMA, collagen 1α1, CaMKIIδ, TNFα, BMP4, MMP9, CXCR4 and Hey2 (Figure 6F). Altogether, our results demonstrate that *Foxm1* expression in cardiomyocytes is critical for age-induced cardiac hypertrophy but it is not required for TAC-induced hypertrophy.

## Discussion

We have previously shown that *Foxm1* is a critical mediator of heart development and cardiomyocyte proliferation. Total ablation of *Foxm1* in *Foxm1^−/−^* mice [11] or conditional deletion in *Nkx2.5-Cre/Foxm1^fl/fl^* mice [10] caused ventricular hypoplasia and embryonic lethality. However, the postnatal regulation of cardiac development by Foxm1 has not been investigated. In the present study, we generated a novel mouse line utilizing the Cre-LoxP system to selectively delete *Foxm1* from cardiomyocytes in the late postnatal period using the α-myosin heavy chain Cre (αMHC-Cre) [15]. *αMHC-Cre/Foxm1^fl/fl^* mice were viable and healthy, displaying no heart abnormalities until old age. Despite a 70% decrease in Foxm1 mRNA and protein at P7, there was no change in the percentage of Ki-67-positive cardiomyocytes in *αMHC-Cre/Foxm1^fl/fl^* hearts. Furthermore, the proliferation of Cre-positive and Cre-negative cardiomyocytes was similar, suggesting that Foxm1 does not regulate cardiomyocyte proliferation in the postnatal heart at P7. Consistent with this hypothesis, we found no differences between control and *αMHC-Cre/Foxm1^fl/fl^* hearts in mRNA levels of cell cycle regulators such as cyclin B_1_, CDC 25B, p21^cip1^ or nMYC. Interestingly, there was a significant decrease in Plk-1, a cell cycle regulator known to be transcriptionally regulated by Foxm1 [10], [24]. Reduced expression of Plk-1 was insufficient to cause proliferation defects in *αMHC-Cre/Foxm1^fl/fl^* hearts. As *αMHC-Cre* expression is low in the early postnatal period (P1-7) [15], [27], our studies do not rule out the possibility that Foxm1 may be important for cardiomyocyte proliferation in the early postnatal period. Our results suggest different requirements for Foxm1 function between embryonic and postnatal cardiomyocytes.

Young adult *αMHC-Cre/Foxm1^fl/fl^* mice had normal cardiac morphology and function; yet old age *αMHC-Cre/Foxm1^fl/fl^* mice had a significant increase in heart weight-to-body weight ratios and cardiomyocyte size as well as myocardial fibrosis. Foxm1 expression has been previously shown to progressively decrease in fibroblasts from adult humans and to be down-regulated in fibroblasts from patients with Progeria syndrome, a rare disorder in which individuals prematurely show signs of aging including cardiovascular disease [8]. We showed here that Foxm1 expression was decreased in the hearts of old mice when compared to young adults. Cardiomyocyte hypertrophy in old *αMHC-Cre/Foxm1^fl/fl^* mice was associated with decreased expression of Hey2, an important transcriptional regulator in cardiomyocytes [13], [26], [28], [29]. We previously showed decreased Hey2 mRNA in *Nkx2.5-Cre/Foxm1^fl/fl^* embryonic hearts which had enlarged cardiomyocytes [10]. These data indicate a genetic link between *Foxm1* and Hey2 that is persistent in the embryonic and adult heart. Since hearts of Hey2-deficient mice exhibited cardiomyocyte hypertrophy [13], decreased Hey2 expression may contribute to cardiac abnormalities in Foxm1-deficient myocardium. Our data also demonstrates that cardiomyocyte-derived Foxm1 is dispensable for cardiac development in the late postnatal period, yet Foxm1 plays a critical role in cardiac maintenance in the older heart.

Given the essential role Foxm1 plays in embryonic cardiac development as well as the propensity for late onset cardiac hypertrophy in mice with cardiomyocyte-specific Foxm1 deletion, we investigated a potential role for Foxm1 in pressure overload-induced cardiac remodeling in young mice. Although aortic constriction increased Foxm1 mRNA and protein in cardiomyocytes of WT mice, there was no difference in cardiac hypertrophy or remodeling between *αMHC-Cre/Foxm1^fl/fl^* and control mice. The gene expression profile was also unaltered between groups following prolonged pressure overload. Thus, Foxm1 expression in cardiomyocytes is not critical for pressure overload-induced cardiac hypertrophy in young mice.

Foxm1 is a known regulator of embryogenesis and has been shown to play tissue- and cell-type specific roles in different organs [7], [14], [19], [30]–[37]. In this study, using *αMHC-Cre/Foxm1^fl/fl^* mice we demonstrated that *Foxm1* is not essential for cardiac development in the late postnatal period into adulthood. Decreased Foxm1 expression at P7 did not alter cardiomyocyte proliferation as did embryonic deletion of Foxm1 from cardiomyocytes [10], suggesting an alternative role for Foxm1 in postnatal compared to embryonic cardiomyocytes. *αMHC-Cre/Foxm1^fl/fl^* mice developed normally into adulthood with normal cardiac morphology and function; however, late in life these mice developed cardiac hypertrophy and fibrosis. These results suggest that Foxm1 is essential for long-term maintenance of cardiac structure and function, as has been previously indicated in patients with Progeria syndrome [8]. It is becoming increasingly evident that cardiomyopathies can manifest at any developmental time point [38]–[41]. In fact, familial hypertrophic cardiomyopathy of known origin can result in strikingly different phenotypes and time of onset within a single family [39]. These findings indicate the heterogeneous nature of the disease and make it evident that multiple factors are involved in disease progression. However, mutations associated with the latest onset of disease phenotype are primarily in genes involved in sarcomere structure and not function [39], [42]. These genes include the myosin binding protein C, troponin I [40], and members of the sarcoglycan family, particularly δ-sarcoglycan [42]. Furthermore, a number of muscular dystrophies have also been shown to present with cardiac abnormalities [3], [4] and defects in other extracellular matrix proteins can result in cardiac aberrations similar to those observed here [4], [5]. Therefore, it is quite possible that Foxm1 may regulate the transcription of a cardiac structural protein or extracellular matrix protein in the heart, the deficiency of which in *αMHC-Cre/Foxm1^fl/fl^* mice may not manifest structural or functional consequences until late in life. Alternatively, Foxm1 may be required for proliferation or differentiation of endogenous cardiac progenitor cells. This hypothesis is consistent with expression of Foxm1 in rare populations of cells in the adult heart. In the absence of Foxm1, cardiac progenitors may not be able to compensate for the normal attrition of cardiomyocytes during the lifetime of the heart. As cells die and cannot be replaced by new cardiomyocytes the existing myocytes compensate by hypertrophying and the deficit between cardiomyocyte loss and existing cardiomyocyte hypertrophy is balanced by scar formation. The absence of Foxm1 may lead to impairment in cardiac maintenance, causing hypertrophy and fibrosis during aging. Despite a propensity for aging-mediated cardiac hypertrophy when cardiomyocyte-derived Foxm1 is absent, Foxm1 is not a mediator of the timeline or extent of cardiac hypertrophy or remodeling following aortic banding.

In summary, Foxm1 is not essential for postnatal cardiac development or cardiomyocyte proliferation after postnatal day 7 but plays an important role in maintenance of cardiac structure during aging. Mice with cardiomyocyte-specific deletion of *Foxm1* develop cardiac hypertrophy and fibrosis late in life. Our results demonstrate that Foxm1 function in cardiomyocytes is dependent on age and disease state.
